# Protracted infusional 5-fluorouracil plus high-dose folinic acid combined with bolus mitomycin C in patients with gastrointestinal cancer: a phase I/II dose escalation study

**DOI:** 10.1038/sj.bjc.6601412

**Published:** 2003-11-25

**Authors:** J T Hartmann, K Oechsle, D Quietzsch, A Wein, R D Hofheinz, F Honecker, O Nehls, C-H Köhne, G Käfer, L Kanz, C Bokemeyer

**Affiliations:** 1 Abt. Hämatologie/Onkologie/Immunologie/Rheumatologie, Medizinische Klinik II, Eberhard-Karls-Universität, Tübingen, Germany; 2 Abt. für Internistische Onkologie, Innere Medizin II, Klinikum Chemnitz, Germany; 3 Medizinische Klinik I, Universität Erlangen-Nürnberg, Germany; 4 Medizinische Klinik III, Klinikum Mannheim, Germany; 5 Abt. Gastroenterologie/Hepatologie/Infektiologie, Medizinische Klinik I, Eberhard-Karls-Universität, Tübingen, Germany; 6 Medizinische Klinik I, Universität Dresden, Germany; 7 Kliniken Sigmaringen, Innere Medizin, Germany

**Keywords:** advanced gastrointestinal cancer, colorectal carcinoma, gastric cancer, mitomycin C, continuous infusional 5-flourouracil, phase I/II study

## Abstract

The aim of this study was to define the maximum tolerated dose (MTD) of bolus mitomycin C (MMC) in combination with 24 h-continuous infusion of 5-flourouracil (FU) plus folinic acid, and to assess the toxicity and activity in patients with previously treated colorectal and gastric cancer. Escalating doses of MMC starting from 6 mg m^−2^ in 2 mg m^−2^-steps to a maximum of 10 mg m^−2^ were applied on days 1 and 22, given to fixed doses of 5-FU (2.600 mg m^−2^) as 24 h infusion and folinic acid 500 mg m^−2^ prior to 5-FU weekly for 6 weeks. At least three patients were treated at each dose level. A total of 16 patients have been included in the phase I study. At the highest dose level (MMC 10 mg m^−2^), grade III thrombocytopenia, dyspnoea, mucositis and diarrhoea were observed in one patient each (17 %). In the phase II study 45 patients, 33 with colorectal cancer and 12 with gastric cancer, 23 patients after failure of first- and 22 patients after at least second-line or subsequent chemotherapy have been treated. Seven partial responses (PR) were registered (16%), one (3%; CI_95%_, 0–16) in colorectal and six (50%; CI_95%_, 21–79%) in gastric cancer patients. In all, 17 (38%) achieved disease stabilisation, 15 colorectal (45%, CI_95%_, 28–64%) and two gastric cancer patients (17%; CI_95%_, 2–48%). The median progression-free survival was 3.1 months (range, 0.9–9.1) in colorectal and 4.6 months (range, 0.7–12.4) in gastric cancer. The median overall survival time was 6.6 months (range, 1.9–15.6) in colorectal and 7.1 months (range, 1.7–20.8) in patients with gastric cancer. This regimen was considered to be safe and well tolerated for pretreated patients with gastrointestinal adenocarcinoma. In gastric cancer,MMC plus infusional 5-FU/folinic acid may be a potential second-line regimen with promising antitumour activity.

Mitomycin C (MMC) is a quinone-containing antitumour antibiotic that is reductively activated by a variety of enzymatic systems to metabolites that alkylate and crosslink DNA (Dorr, 1988). The treatment with MMC results in the formation of reactive oxygen species, which also contribute to its cytotoxicity (Doroshow, 1986; Pritsos and Sartorelli 1986; Dusre *et al*, 1990). Intracellular glutathione (GSH) participates in the detoxification of alkylating agents by converting reactive intermediates into noncytotoxic metabolites, and cells with increased pools of GSH have been shown to develop resistance to MMC (Dorr, 1988).

Single-agent MMC reaches remission rates of approximately 10–20% in patients with advanced gastrointestinal cancer, in first-line as well as in second-line therapy (Moertel *et al*, 1968; Moertel, 1975). In patients refractory to 5-flourouracil (FU) /folinic acid, first-line chemotherapy single-agent MMC has shown antitumour efficacy (Hartmann *et al*, 1998a; Hartmann *et al*, 1999). Partial remissions have been found particularly in gastric cancer, as well as disease stabilisations in colorectal cancer patients. When combined with continuous infusional 5-FU/folinic acid, an increased antitumour activity was found in pretreated gastrointestinal adenocarcinomas with objective remission rates of about 10% in colorectal and up to 46% in gastric cancer patients (Chester *et al*, 2000; Kretzschmar *et al*, 2000; Hofheinz *et al*, 2002). Different schedules and dosages of MMC have been used in previous trials (Ross *et al*, 1997; Kretzschmar *et al*, 2000; Chang *et al*, 2002; Hofheinz *et al*, 2002; Tebbutt *et al*, 2002), but no systematic investigation to determine the maximum tolerated dose (MTD) of MMC when combined with 5-FU/folinic acid has been performed so far.

The aim of this nonrandomised multicentre phase I/II study was first to define the MTD of bolus MMC in combination with continuous infusional 5-FU plus high-dose folinic acid. In the phase II part, the question of antitumour activity was addressed adding MMC to 5-FU/folinic acid in 5-FU-resistant patients with metastatic gastrointestinal adenocarcinomas.

## PATIENTS AND METHODS

### Eligibility

Patients eligible for this study had advanced disease of a histologically confirmed gastrointestinal adenocarcinoma. In the phase I part of this study, patients were allowed to be pretreated with any palliative chemotherapy. Objective evidence of tumour progression must have occurred while the patient was receiving chemotherapy or within 6 months from receiving the last dose of prior chemotherapy. Disease must have been bidimensional measurable in X-ray, ultrasound or computed tomography.

In the phase II part of the trial, patients were allowed to have up to two prior 5-FU-based chemotherapy regimens, bolus and/or continuous infusion of 5-fluorouracil/folinic acid. Adjuvant chemotherapy was also permitted. Patients must have been between 18 and 75 years, have a performance status of 0–2 according to the ECOG criteria and a life expectancy of at least 3 months. Other inclusion criteria consisted of pretreatment neutrophile count ⩾2000 *μ*l^−1^, platelet count ⩾150 000 *μ*l^−1^, serum creatinine concentration ⩽2.0 mg dl^−1^ and total bilirubin levels ⩽2.0 mg dl^−1^. Exclusion criteria were known incompatibility to 5-FU or MMC, the presence of central nervous metastases, serious or uncontrolled concurrent medical illness or secondary malignancies except for *basal cell carcinoma of the skin* or carcinoma *in situ* of cervix uteri. All patients were informed of the investigational nature of this study and had to provide written informed consent in accordance with institutional and federal guidelines. The multicentre study was reviewed and approved by the local ethical committee of all participating centres. Treatment was performed according to the Declaration of Helsinki.

### Treatment protocol

Chemotherapy consisted of 7-weekly courses of 2600 mg m^−2^ 5-FU i.v. as a 24-h continuous infusion combined with 500 mg m^−2^ folinic acid i.v. applied over 2 h or simultaneously in one infusion bag as a 24-h infusion with 5-FU (Oncolfolic®, Medac, Hamburg, FRG), both given weekly for 6 weeks followed by 1 week rest. The treatment was restarted on day 50. Mitomycin C was administrated as bolus injection in three dose escalation steps of 6, 8 and 10 mg m^−2^ on days 1 and 22. At least three patients were treated at each dose level in the first part (phase I). Treatment was continued for at least two treatment cycles. Escalation proceeded to dose level 3 unless two out of three or four out of six patients experienced a dose-limiting toxicity (DLT) after the first cycle. Treatment was applied in an outpatient oncology setting. All patients prophylactically received 50 mg of prednisolone i.v. before mitomycin application. Antiemetic premedication was left up to the decision of the treating physician. Most patients received 5-HT_3_-antagonists or metoclopramide prior to treatment.

### Evaluation of response and toxicity

All patients were assessed prior to treatment by physical examination, routine haematology and biochemistry analysis, chest X-ray and CT scan to define the extent of disease. Complete blood cell counts with platelets and differential counts were obtained weekly during chemotherapy, and serum chemistry analyses were repeated at least once every course. Subjective symptoms, physical examination, performance status and all adverse reactions were recorded before each treatment cycle. Tumour size was measured every cycle by CT scan, X-ray or any other technique that allows retrospective and independent assessment.

Determination of tumour response followed WHO standard criteria: complete remission (CR) was defined as a complete disappearance of all evidence of disease. A partial response (PR) was defined as radiological response >50% in tumour size, and response <50% or progression <25% was defined as stable disease. Progressive disease was defined as either residual lesions increasing in size or occurrence of new lesions.

Treatment was continued until one of the following criteria was met: development of progressive disease or of unacceptable toxicity; intercurrent, noncancer-related illness that prevented continuation of therapy or regular follow-up evaluation; withdrawal of consent. The duration of response was defined as the interval from the onset of response until evidence of disease progression. Overall survival was defined as the interval from the date of study until death (or last contact if the patient was still alive).

Dose-limiting toxicity was defined as grade IV haematological toxicity (neutrophile granulocyte, thrombocytes), diarrhoea grade III, febrile neutropenia grade III with any grade of diarrhoea or any organ toxicity grade III (nephrotoxicity grade II), except alopecia and nausea. Dose modifications for the following treatment cycles were based on the worst toxicity observed during the previous cycle of chemotherapy. Treatment was delayed in case of leucocytes <3000 *μ*l^−1^ or thrombocytes <75000 *μ*l^−1^ to a maximum length of 2 weeks.

### Statistical analysis

All information was included into a database at Tuebingen University Medical Center, Germany. Statistical analyses were performed using the SPSS system (SPSS for windows 10.0 software, Microsoft Corp., Redmond, WA, USA). Exact 95% confidence intervals (CI) around the observed response rate were calculated from the binominal distribution. The Kaplan–Meier method was used to determine overall and progression-free survival distributions. The overall survival calculation used death due to any reason as the endpoint (Kaplan and Maier, 1958).

## RESULTS

Between September 1999 and May 2002, a total of 61 patients with advanced gastrointestinal cancer failing 5-FU-based chemotherapy were included according to the study protocol.

### Phase I

A total of 16 patients were entered into the phase I part and all three planned consecutive dose levels, including 6, 8 and 10 mg m^−2^ of bolus MMC, have been tested. At the highest dose level (10 mg m^−2^ mitomycin) grade III thrombocytopenia, dyspnoea, mucositis and diarrhoea were observed in one patient each (17%). No grade IV toxicity was seen in this phase I part of the study and no grade III toxicity occurred at the dose levels of 6 or 8 mg m^−2^ MMC. Therefore, 10 mg m^−2^ was considered to be the MTD of mitomycin given on days 1 and 22 in combination with weekly infusional 5-FU plus folinic acid. Toxicity according to WHO criteria at dose level 3 during the phase I study is listed in Table 1

**Table 1:** Toxicity at dose level 3 (10 mg m^−2^ MMC) according to the WHO criteria in the phase I (*n*=6 patients; *n*=9 cycles)

**Definition of toxicity**	**Grade I/II (%)**	**Grade III (%)**	**Grade IV (%)**
Neutropenia	2 (33 %)	0	0
Thrombocytopenia	2 (33 %)	1 (17%)	0
Anemia (nonhaemolytic)	2 (33%)	0	0
Nausea	1 (17%)	0	0
Emesis	1 (17%)	0	0
Diarrhoea	1 (17%)	1 (17%)	0
Constipation	2 (33%)	0	0
Dyspnoea	0	1 (17%)	0
Mucositis	1 (17%)	1 (17%)	0
Hand–foot syndrome	1 (17%)	0	0

.

### Phase II

#### Patients' characteristics

In total, 45 patients, 37 men and 8 women, were entered into the phase II study. All were assessable for response evaluation, survival analysis and toxicity. A summary of baseline patients' characteristics is given in Table 2

**Table 2:** Characteristics of patients treated at dose level 3 (10 mg m^−2^ MMC) during the phase II study (*n*=45 patients)

	***N* (patients)**	**%**
Sex	37	82
Male		
Female	8	18
Median age	59 years (range, 44–73)	
Performance status (ECOG)		
0	12	27
1	27	60
2	6	13
Site of primary tumour		
Colon carcinoma	11	24
Rectal carcinoma	22	49
Gastric carcinoma	12	27
Sites of metastases		
Liver	35	78
Lymph node	16	36
Lung	20	44
Locoregional recurrence	15	33
Bone	2	4
Other	7	16
No. of previous chemotherapy regimens		
*n*=1	23	51
*n*⩾2	22	49
Prior chemotherapeutic regimens in gastric cancer (*n*=12)		
c.i. 5-FU	6	50
c.i. 5-FU combined with cisplatin	4	33
Other	2	22
First-line chemotherapeutic regimen in colorectal cancer (*n*=33)		
Bolus 5-FU	9	27
c.i. 5-FU	15	46
c.i. 5-FU combined with irinotecan	3	9
c.i. 5-FU combined with oxaliplatin	6	18

c.i.=continuous infusion.

.

In all, 11 patients (24%) had colon, 22 (49%) rectal and 12 (27%) had gastric adenocarcinomas. The majority of patients were symptomatic, with 27 patients (60%) having an ECOG-performance status of 1 and six patients (13%) of 2. A total of 23 patients (51%) were included after failure of first- and 22 patients (49%) after at least one further line of chemotherapy. In all, 50% of the patients (*n*=6) with gastric cancer had been pretreated with continuous infusional 5-FU as prior treatment and 33% of the patients (*n*=4) had received 5-FU-combination chemotherapy with cisplatin. In patients with colorectal cancer, previous chemotherapy consisted of bolus 5-FU in 27% (*n*=9) and continuous infusion of 5-FU in 46% (*n*=15). A total of 27% (*n*=9) had received continuous 5-FU plus either irinotecan or oxaliplatin.

### Safety and toxicity assessment

A median number of two cycles of MMC and 5-FU/folinic acid (range, 1–3) have been applied during the phase II trial. The combination of continuous infusional 5-FU plus folinic acid and bolus MMC was well tolerated. The side effects observed in the phase II are listed in Table 3

**Table 3:** Worst toxicity per patient during treatment with 5-FU/folinic acid and MMC in phase II (*n*=45 patients; *n*=85 cycles)

**Definition of toxicity**	**Grade I/II (%)**	**Grade III (%)**	**Grade IV (%)**
Anaemia	25 (55 %)	3 (7 %)	0
Leucocytopenia	21 (47 %)	3 (7 %)	0
Thrombocytopenia	17 (38 %)	7 (16 %)	0
Infection	3 (7 %)	2 (4 %)	0
Diarrhoea	15 (33 %)	7 (16 %)	0
Mucositis	11 (24 %)	2 (4 %)	0
Constipation	7 (16 %)	0	0
Emesis	22 (49 %)	3 (7 %)	0
Nausea	16 (36 %)	3 (7 %)	0
Hand–foot –syndrome	9 (20 %)	0	0

.

No renal dysfunction, pulmonary toxicity such as irreversible interstitial *pneumonia* or evidence of haemolytic uraemic syndrome or haemolytic anaemia was observed. The most frequent severe haematological toxicity (grade III/IV according to the WHO criteria) during the phase II trial was thrombocytopenia in 16% of the patients (*n*=7). In three of those seven patients, thrombocytopenia occurred during the first cycle, and in the remaining four patients in the second cycle. Treatment has to be delayed for 2 weeks in one single patient. One of seven patients had thrombopenia grade II prior to the start of treatment with MMC/5-FU due to pretreatment. Patients with severe thrombopenia regenerated slowly after withdrawal of treatment. Mild thrombocytopenia was more frequent (*n*=17, 38%). Leucocytopenia grade I/II occurred in 47% of the patients (*n*=21), and grade III/IV was seen in 7% of the patients (*n*=3). In all, 4% of the patients (*n*=2) experienced severe neutropenic infections. Nonhaemolytic anaemia grade III occurred in 7% of the patients (*n*=3). These patients all had mild anaemia prior to the study. Diarrhoea was the most frequent nonhaematologic grade III/IV toxicity in 16% of the patients (*n*=7), followed by nausea and emesis in 7% (*n*=3). Mild hand–foot syndrome was observed in 20% of the patients (*n*=9). No treatment-related death was seen and no catheter-related complications were observed. The treatment dosage had to be reduced in 11% of the patients (*n*=5) due to toxicity. Treatment was delayed for at least 1 week during the whole treatment in 20 patients (44%) due to toxicity. Seven patients (16%) were hospitalised as a result of toxicity in seven of 85 cycles (8%).

### Response evaluation

Seven patients (16%) attained an objective response to the combination of infusional 5-FU plus folinic acid and MMC, one of 33 patients with colorectal and six of 12 patients with gastric carcinoma. This represents a 50% response rate (CI_95%_ 21–79) for the 12 patients with gastric adenocarcinoma, and a 3% response rate (CI_95%_ 0–16) in colorectal cancer patients. No complete response was seen. Disease stabilisations were achieved in 17 patients (38%), 15 with colorectal cancer (45%, CI_95%_ 28–64) and two with gastric cancer (17%, CI_95%_ 2–48). In all, 17 patients with colorectal cancer (52%, CI_95%_ 33–69) and four patients with gastric cancer (33%, CI_95%_ 10–65) had progressive disease at the first tumour evaluation on treatment with MMC/-5-FU.

The median progression-free survival was 3.1 months (range, 0.9–9.1) in patients with advanced colorectal cancer and 4.6 months (range, 0.7–12.4) in gastric cancer patients. The median overall survival in patients with colorectal cancer was 6.6 months (range, 1.9–15.6) and 7.1 months in patients with gastric cancer (range, 1.7–20.8). Patients responding to treatment and those with tumour stabilisation achieved a median survival of 9.5 months (range, 3.8–20.8), whereas in patients with progressive disease the median survival was 5.3 months (range, 1.7–12.4).

The 23 patients with one previous line of chemotherapy prior to the start of 5-FU/folinic acid plus MMC had a median survival of 8.4 months (range, 1.6–18.3), and the 22 patients with at least two previous chemotherapy regimen survived for only 5.9 months (range, 1.3–15.6). Of the patients, 14 (61%) with second-line 5-FU/folinic acid plus MMC treatment achieved at least disease stabilisation and 10 patients (39%) had progressive disease, while in patients ⩾2 previous regimens, only 45% (*n*=10) responded to treatment and 55% (*n*=12) had progressive disease. Patients with gastric cancer developing progressive disease while receiving previous chemotherapy had a lower probability to attain an objective response compared to the subgroup of patients with progressive or recurrent disease within 6 months of receiving the last dose of previous chemotherapy (*P*=0.02) (see Table 4

**Table 4:** Response to infusional 5-FU/folinic acid plus MMC depending on the progression-free interval during prior chemotherapy

**Response to MMC/FU/FS**	**PD during pretreatment**	**PD after PFI**	**N.e.**	**Total**
PR/NC	−/2	5/−	1/−	8
PD	3	1	—	4
Total	5	6	1	12

χ^2^, *P*=0.02. MMC=mitomycin C; FU=5-fluorouracil; FS=folinic acid; PR=partial remission; NC=no change; PD=progressive disease; PFI=progression-free interval ; n.e.=not evaluable.

).

## DISCUSSION

The prognosis of patients with recurrent metastatic gastric cancer is dismal, with a median survival ranging between 3 and 5 months in untreated patients (Glimelius *et al*, 1997). In advanced gastric cancer, first-line use of palliative combination chemotherapy compared with best supportive care is superior in terms of quality of life and overall survival. Whether this is also true for patients with relapse after first-line chemotherapy is currently not proven. Comparable to the treatment strategies in colorectal cancer, in recent years, several attempts have been made to improve the results by introducing continuous 5-FU infusion into polychemotherapy regimens in this disease. Mitomycin C is among the most effective single agents for the treatment of advanced gastric cancer (Schnall and Macdonald, 1993).

Although second-line chemotherapy in gastric cancer is not considered as a standard approach, phase II data are available for 5-FU/folinic acid, docetaxel, fotemustine and paclitaxel, demonstrating objective remission rates in a range of 0–22% and overall survival times ranging from 3 to 8 months (Vanhoefer *et al*, 1994; Di Bartolomeo *et al*, 1996; Rougier *et al*, 1996; Cascinu *et al*, 1998; Wilke *et al*, 1998). By using continuous infusional MMC as a single agent, 30% of pretreated gastric cancer patients will achieve an objective remission. However, the median survival time is short with approximately 3–4 months (Hartmann *et al*, 1998a; Hartmann *et al*, 1999).

The cellular mechanism of the clinically observed synergy between MMC and infusional 5-FU has not been further elucidated, although time-dependent interactions between both drugs on human cancer cell lines have been reported (Russello *et al*, 1989). In previous trials, MMC was applied either using absolute doses (e.g. 15 mg; Chang *et al*, 2002) or in relation to body surface with 7–10 mg m^−2^ on a 3-weekly basis. However, a systematic investigation to determine the MTD of MMC when combined with 5-FU/folinic acid has not been performed so far (Ross *et al*, 1997; Kretzschmar *et al*, 2000; Hofheinz *et al*, 2002; Tebbutt *et al*, 2002). With different schedules – one single injection of 15 mg m^−2^ mitomycin on day 1 (Kretzschmar *et al*, 2000) or by combining infusional 5-FU with folinic acid with 10 mg m^−2^ MMC every 3, respectively every 6, weeks (Hofheinz *et al*, 2002; Tebbutt *et al*, 2002) – survival in gastric cancer patients ranging from 5.3 to 10.2 months has been reported. Objective remission rates between 37 and 51% have been observed (Kretzschmar *et al*, 2000; Hofheinz *et al*, 2002; Tebbutt *et al*, 2002). In the phase I part of the present study, the MTD of MMC when combined to weekly infusional 5-FU plus folinic acid was 10 mg m^−2^ at days 1 and 22, and the rate of partial responses in the phase II was 50% lasting for a median of 3.5 months in pretreated gastric cancer patients. Furthermore, 31% of the patients achieved disease stabilisation. The overall survival for these patients was 7.1 months; not significantly longer compared to investigations of other agents as second-line treatment. This demonstrates the aggressive biology of the disease with a short interval between progression after chemotherapy and death.

Several trials have demonstrated that MMC also displays some activity in previously treated patients with colorectal cancer (Moertel *et al*, 1968; Conti *et al*, 1995; Hartmann *et al*, 1998a). A recent phase III trial in advanced colorectal cancer comparing protracted infusion of 5-FU with or without the addition of MMC demonstrated an overall response rate of 45% for the combination and 38% for 5-FU alone. The failure-free survival was 7.9 months in favour of the combination compared to 5.4 months for 5-FU alone. In addition, the global quality of life scores at 6 months was better for 5-FU and MMC (Ross *et al*, 1997). In the second-line setting, a considerable number of trials using MMC plus continuous infusional 5-FU have been carried out demonstrating that the addition of MMC to 5-FU can overcome resistance to first-line chemotherapy in a distinct number of patients (Conti *et al*, 1995; Becker *et al*, 1997; Seitz *et al*, 1998; Chester *et al*, 2000; Tassinari *et al*, 2000; Grumett *et al*, 2001; Ross *et al*, 2002).

In the present investigation, using MMC at maximum doses in combination with infusional 5-FU, a low remission rate of 3% was observed. However, 45% of patients revealed disease stabilisation lasting for a median interval of 3.2 months. These data are clearly inferior to other agents such as irinotecan (CPT-11) (Rothenberg *et al*, 1996; Rougier *et al*, 1997; Van Cutsem *et al*, 1999) or oxaliplatin plus 5-FU/folinic acid (de Gramont *et al*, 1997), and appear equal to infusional 5-FU plus high-dose folinic acid (Ardalan *et al*, 1991; Weh *et al*, 1994; Hartmann *et al*, 1998b) alone. Thus, with currently available treatment options for metastatic colorectal cancer, a clear role for infusional 5-FU/bolus MMC cannot be identified (Hejna *et al*, 2000; Comella *et al*, 2001; Sobrero *et al*, 2001; Scheithauer *et al*, 2002).

In conclusion, infusional 5-fluorouracil plus folinic acid combined with 10 mg m^−2^ bolus MMC is a safe and well-tolerated regimen that can be given on an outpatient basis. Toxicity was generally mild and treatment was well tolerated. Despite other reports, we did not observe adverse events such as a haemolytic uraemic syndrome, severe pulmonary toxicity, nephrotoxicity or microangiopathic haemolytic anaemia The median dose of MMC applied was 34.7 mg m^−2^ ranging from 17.5 to 61.8 mg m^−2^. Thus, a relationship between the cumulative dose of MMC applied and the risk of developing haemolytic uraemic syndrome remains unclear (Becker *et al*, 1997). However, all patients in the present trial prophylactically received 50 mg prednisolone prior to MMC (Hartmann *et al*, 1998a, 1999, 2000). Treatment delays were mainly due to cumulative thrombocytopenia. While no substantial efficacy was seen in pretreated patients with colorectal cancer, this regimen possess clear antitumour activity in patients with pretreated gastric cancer, particularly in patients with temporary disease stabilisation after completion of preceding chemotherapy.
